# Supplementary material for: Supporting cells orchestrate noise-induced hearing loss via a Gasdermin D-dependent signaling loop with hair cells
Source: Nat Commun. 2025 Dec 17;16:11181. doi: 10.1038/s41467-025-66152-6 (PMC12712061; doi:10.1038/s41467-025-66152-6)
Supplement: Supplementary file 1 — Supplementary Information [file 41467_2025_66152_MOESM1_ESM.pdf]

Supplementary Materials for

Supporting cells orchestrate noise-induced hearing loss via Gasdermin D-dependent signaling loop with hair cells

The PDF file includes: Supplementary Fig. 1 to 13 and Table 1 to 2.

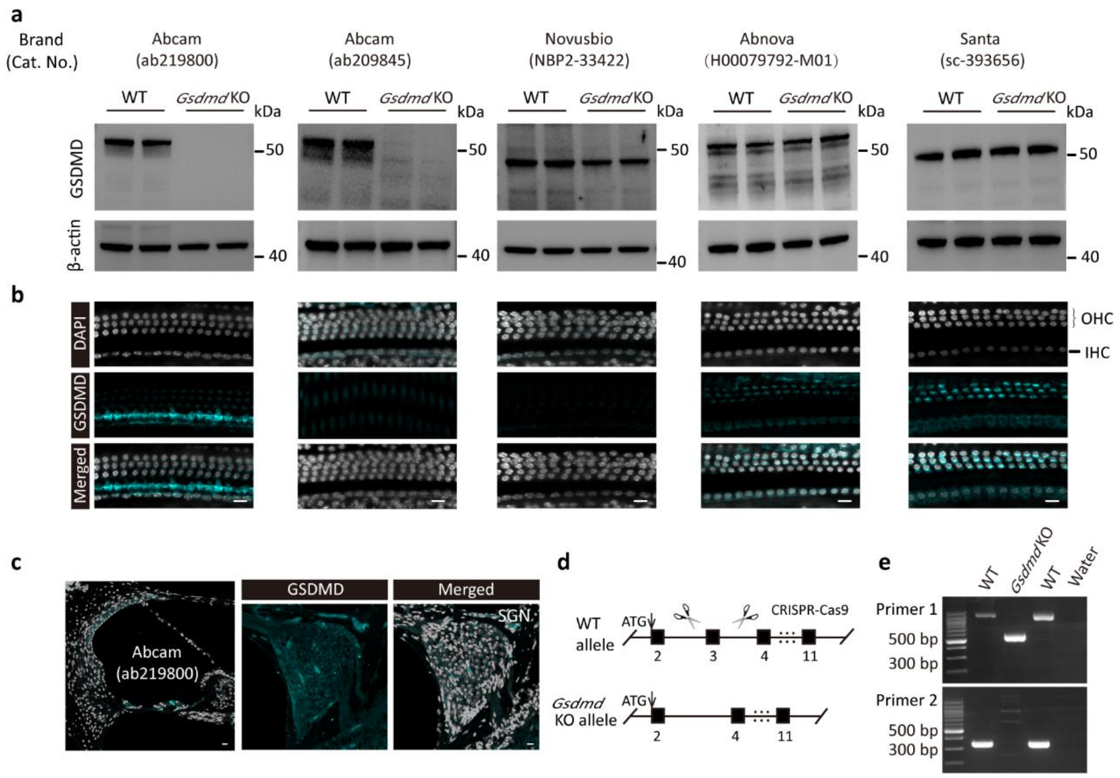

Supplementary Fig. 1: Validation of GSDMD antibodies and *Gsdmd* KO allele.

a WB analysis confirmed that only two of five commercially available antibodies (Abcam, ab219800, and ab209845) were validated for use in cochlear samples from *Gsdmd* KO mice. **b** Representative confocal immunofluorescence images showing GSDMD detection using the five commercial antibodies. **c** Immunolabeling of GSDMD in the organ of Corti and spiral ganglion neurons (SGN). **d** Schematic diagram illustrating the construction of *Gsdmd* KO mice. **e** Agarose gel image of tail-DNA PCR shows a 568 bp band corresponding to the *Gsdmd* KO allele when amplified with Primer 1, while no band is detected with Primer 2. WT: wild type. Scale bar = 10 μ m.

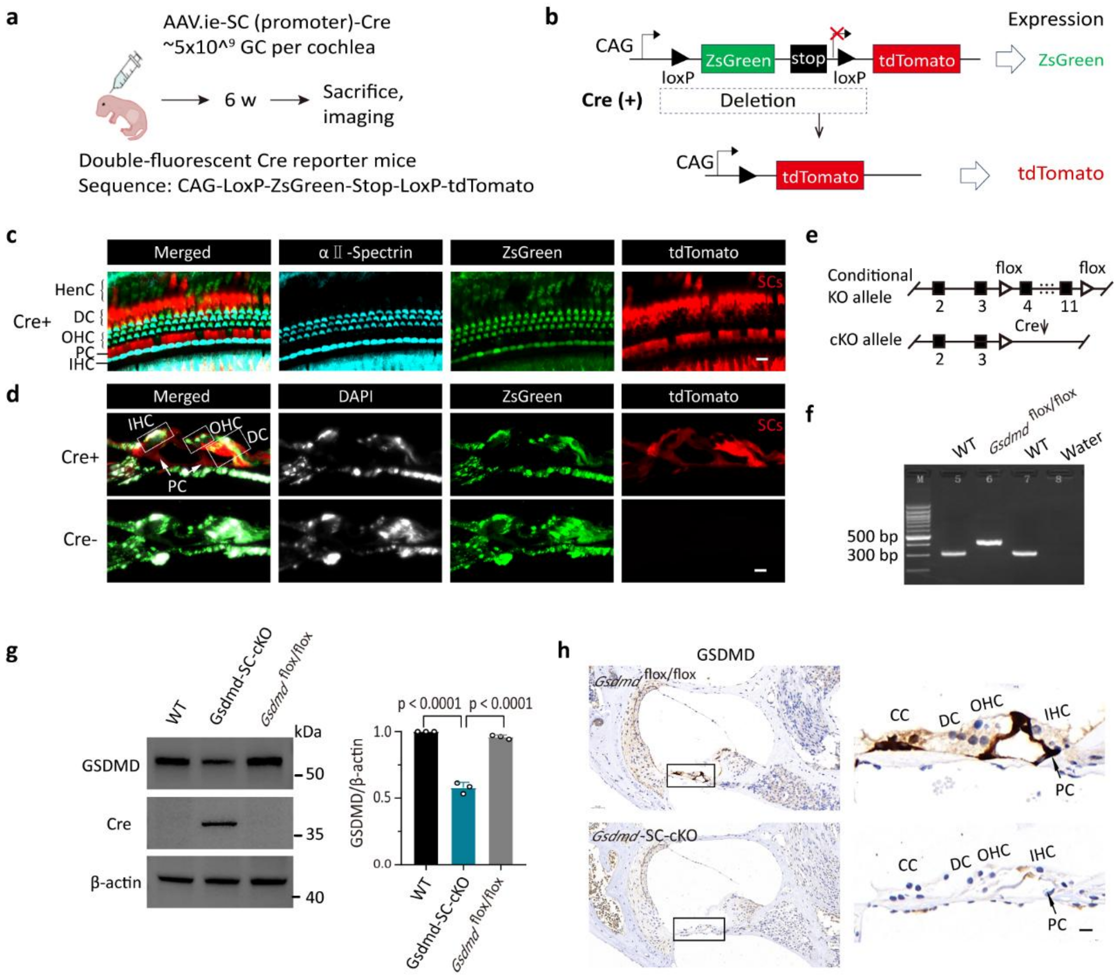

Supplementary Fig. 2: AAV.ie loaded specific promoter mediates Cre-driven gene conditional knockout in SCs

a Schematic diagram of the experimental design. AAV.ie-SC-Cre was injected into the cochlea of reporter mice (CAG-LoxP-ZsGreen-Stop-LoxP-tdTomato). Cre expression driven by the SC-specific promoter excised the ZsGreen cassette and initiated tdTomato expression, indicating the feasibility of the AAV.ie-SC-Cre system. **c** Immunofluorescence images of whole-mount cochlear preparations from reporter mice transfected with AAV.ie-SC-Cre. HCs were labeled with αII-Spectrin (cyan). **d** Cross-section of cochleae from adult mice (6 w-old-age), showing tdTomato expression in AAV-transfected SCs, but absent in HCs. **e** Schematic diagram illustrating the

generation of *Gsdmd* conditional knockout (cKO) mice. Agarose gel image of tail-DNA PCR showing a of 339 bp band for the WT allele and 444 bp band for the *Gsdmd* $^{flox/flox}$ allele. **f, g** WB analysis confirmed significant GSDMD knockdown in the cochleae of *Gsdmd* $^{flox/flox}$ mice transfected with AAV.ie-SC-Cre. Data are presented as means ± SEM (n = 3 biological replicates). Statistical analysis was performed using one-way ANOVA with Bonferroni post-hoc test. **h** Immunohistochemical staining of GSDMD in cochlear sections from WT and *Gsdmd* KO mice, with a magnified view showing its expression in the organ of Corti. Scale bar = 10 μ m. GC: genome copies. WT: wild type. OHC: outer hair cell. IHC: inner hair cell. PC: pillar cell. DC: Deiters' cell. HenC: Hensen's cell. SC: supporting cell. CC: Claudius' cell.

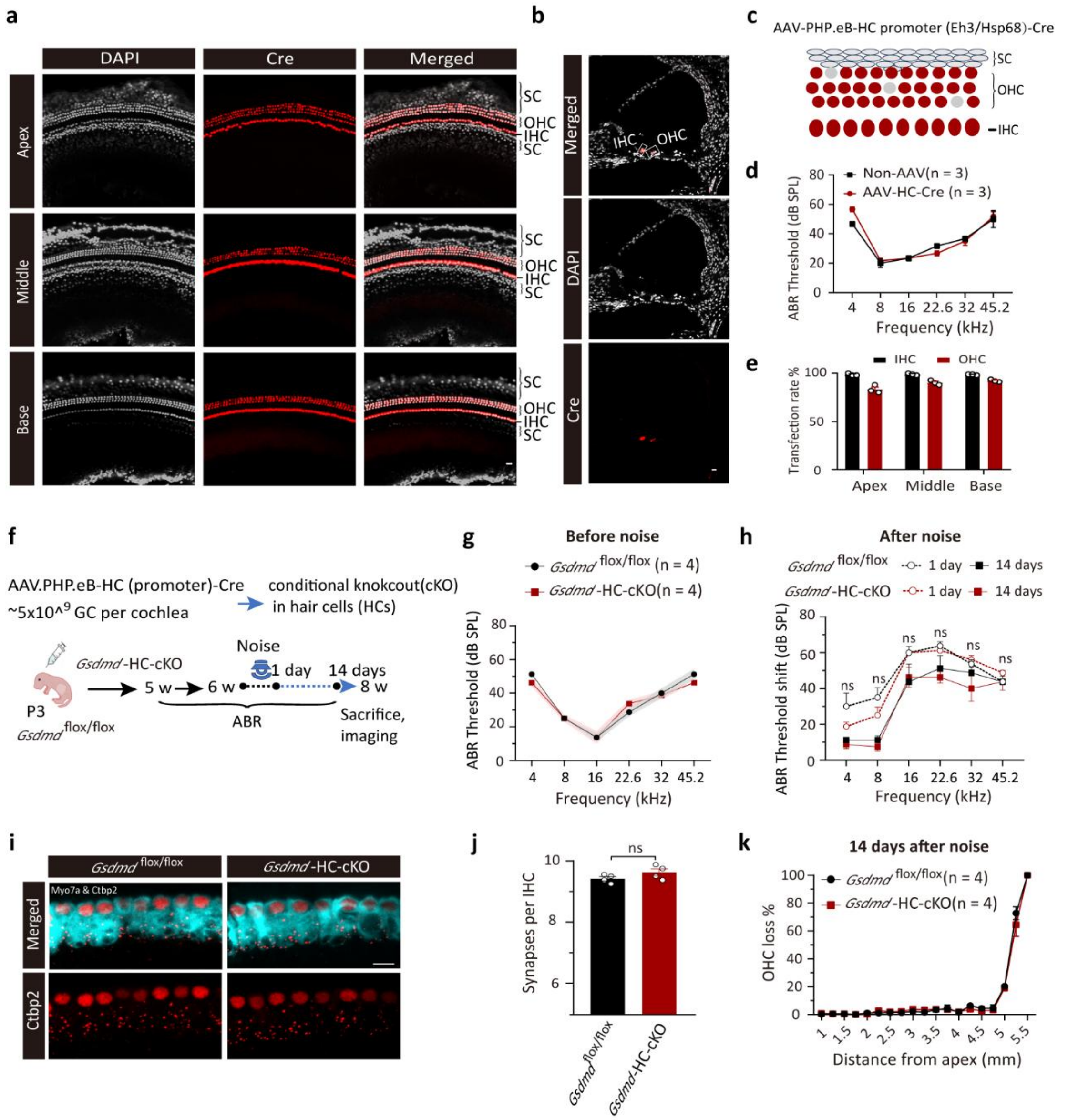

Supplementary Fig. 3: *Gsdmd* conditional knockout in HCs did not prevent noise-induced cochlear damage

a, b, c Cre is specifically expressed in HCs via the AAV.PHP.eB vector loaded with the HC promoter (*Rbm24*-Eh3 sequence following by *Hsp68* mini-promoter). **d** Evaluation of auditory function in mice with AAV.PHP.eB-HC-Cre injection and its transfection efficiency in HCs (> 95% for IHCs across all frequency range; 83%, 90%, and 92% for OHCs at the apical, middle, and basal cochlear regions, respectively). **f** Schematic of the experimental design. AAV.PHP.eB-HC-Cre (5 x 10^9 GCs) was injected into *Gsdmd*

$^{flox/flox}$ mice to generate *Gsdmd*-HC-cKO mice. **g** Baseline ABR thresholds of *Gsdmd* $^{flox/flox}$ (black) and *Gsdmd*-HC-cKO (red) mice before noise exposure (n = 4). **h** No significant difference in ABR threshold shifts was observed between the *Gsdmd*-HC-cKO (red solid line) and *Gsdmd* $^{flox/flox}$ group (black solid line) at 14 days post-noise exposure. **i** Representative confocal images of Myo7a (cyan) and Ctbp2 (red) staining in the 32 kHz cochlear regions. **j** Quantification of the average number of Ctbp2 puncta per IHC at 32 kHz (eight IHCs per cochlea), with circles representing individual values. **k** Morphological analysis of OHC loss in mice from panel **(h)**. Data are presented as mean ± SEM. Statistical analyses were conducted using two-way ANOVA with Bonferroni's post hoc test (**d, g, h, k**) and unpaired Student's t-test (**j**). Scale bar = 10 μ m. ns, not significant. (p value > 0.05). SC: supporting cell. HC： hair cell. OHC: outer hair cells. IHC: inner hair cells. GC: genome copies.

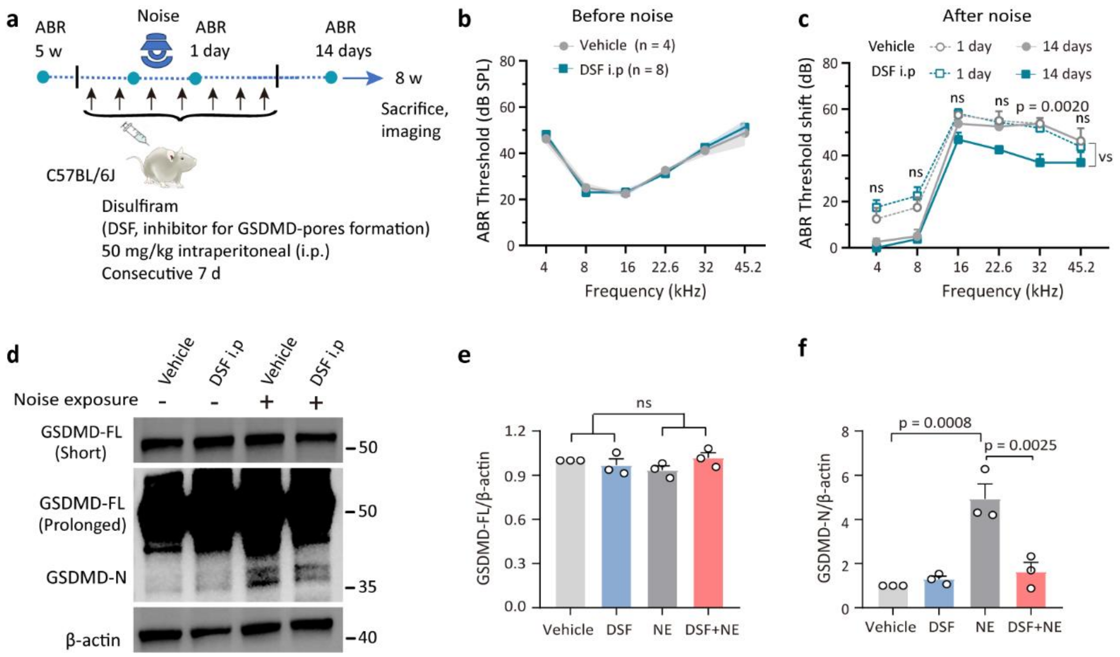

Supplementary Fig. 4: Disulfiram (DSF) attenuates noise-induced hearing dysfunction and GSDMD cleavage

a Schematic of DSF administration and auditory evaluation in the NIHL mice model. **b** Baseline ABR thresholds of the vehicle and DSF groups before noise exposure. **c** ABR threshold shifts recovered at the 32 kHz region in the DSF i.p. group 14 days post-noise exposure (14 d-PNE, cyan solid line) compared to 1 d-PNE values (cyan dashed line), while no recovery was observed in the vehicle groups (gray line). **d** WB analysis of GSDMD-FL and GSDMD-N levels in vehicle and DSF groups with/without noise exposure. Cochleae from noise-exposed mice were collected at 8 h-PNE. Detection was performed with short exposure (5 s) for GSDMD-FL and prolonged exposure (30 s) for GSDMD-N visualization. Data are presented as mean ± SEM (n = 3 biological replicates). Statistical analyses were conducted using two-way (**b, c**) and one-way (**e, f**) ANOVA with Bonferroni's post hoc test. ns, not significant (p value > 0.05). NE: noise exposure. DSF: Disulfiram. i.p.: intraperitoneal.

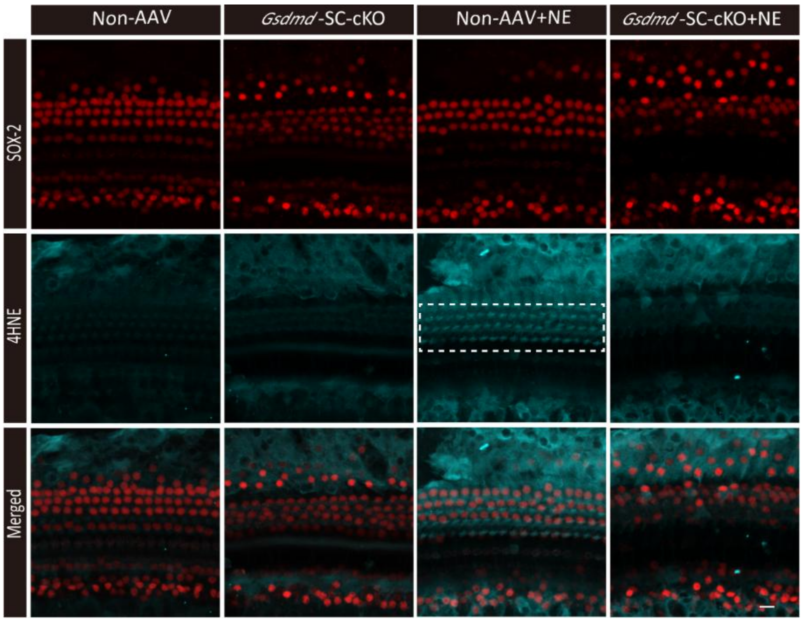

Supplementary Fig. 5: Conditional knockout of *Gsdmd* in SCs alleviated noise-induced 4-HNE accumulation in HCs. Images showed that Sox2-positive SCs (red) and 4HNE-labeled HCs (the region indicated by the dotted square) do not overlap. Scale bar = 10 μ m.

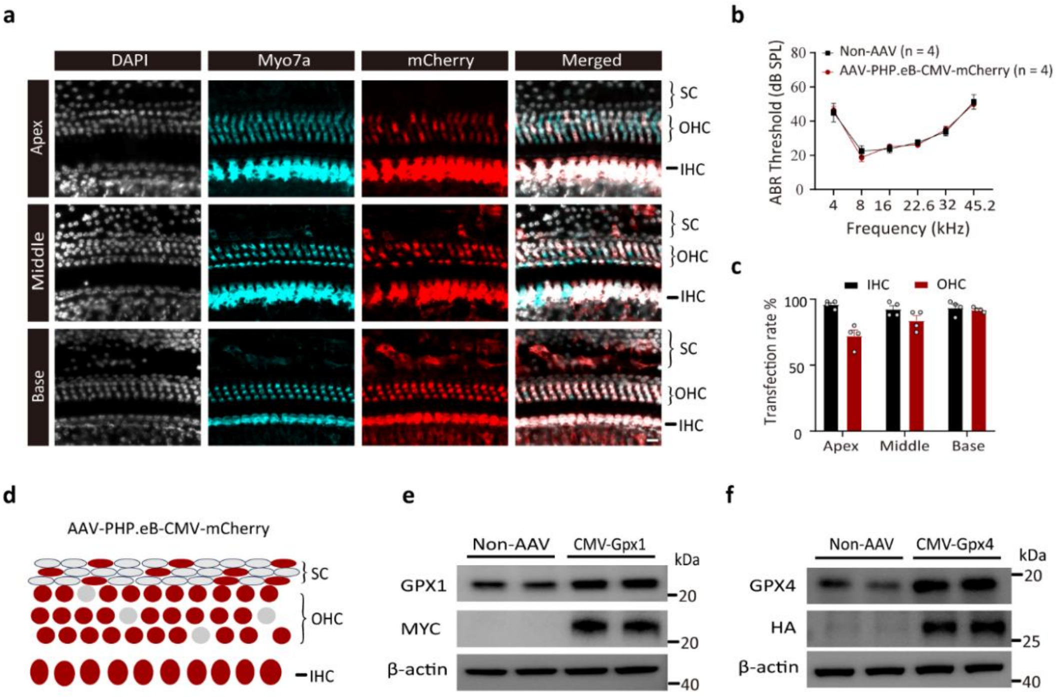

Supplementary Fig. 6: AAV.PHP.eB mediats GPX1 and GPX4 overexpression

driven by CMV promoter in cochlea

a Representative image showing mCherry (red) expression driven by the CMV promoter in AAV.PHP.eB vector. HCs are labeled with Myo7a (cyan). **b** Baseline ABR thresholds showed no significant differences between non-AAV and AAV.PHP.eB-CMV-injected mice. Data are presented as means ± SEM and were analyzed using two-way ANOVA with Bonferroni post hoc test**.** **c** Transduction efficiency of AAV-PHP.eB-CMV in HCs (> 95% for IHCs across all frequency range; 72%, 84%, and 92% for OHCs at the apical, middle, and basal cochlear regions, respectively). **d** Schematic illustrating the transfection efficiency of AAV-PHP.eB vector loading the CMV promoter, showing scattered transfection in SCs. **e, f** WB analysis confirming AAV-mediated overexpression of GPX1 and GPX4 in the cochlea, tagged with MYC and HA, respectively. Cochleae were collected from adult mice (6-week-old). Data are presented as mean ± SEM (n = 4 biological replicates). Statistical analyses were conducted using two-way (**b**) with Bonferroni's post hoc test. SCs: supporting cells. OHC: outer hair cells. IHC: inner hair cells. Scale bar = 10 μ m.

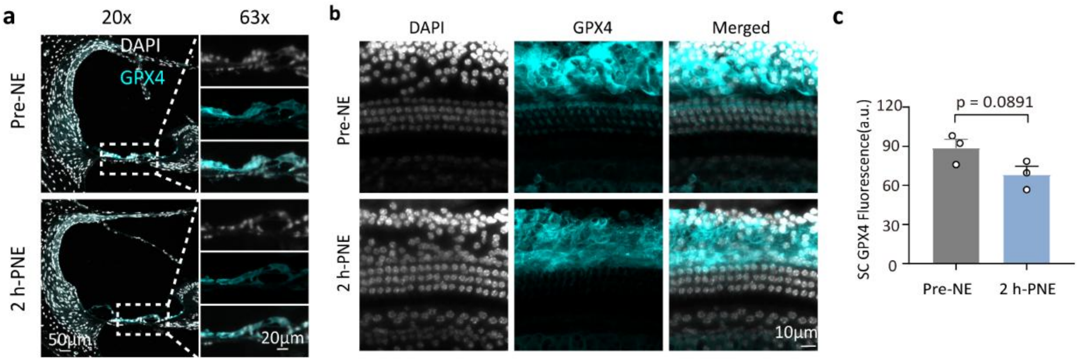

Supplementary Fig. 7: GPX4 expression in cochlea after noise exposure

a, b Representative images showing GPX4 expression (cyan) in the cochlea before and

2 hours after noise exposure. **c** Quantitative analysis of GPX4 immunolabeling in SCs. Data are presented as means ± SEM (n = 3 biological replicates) and were analyzed using unpaired Student's t-test**.**

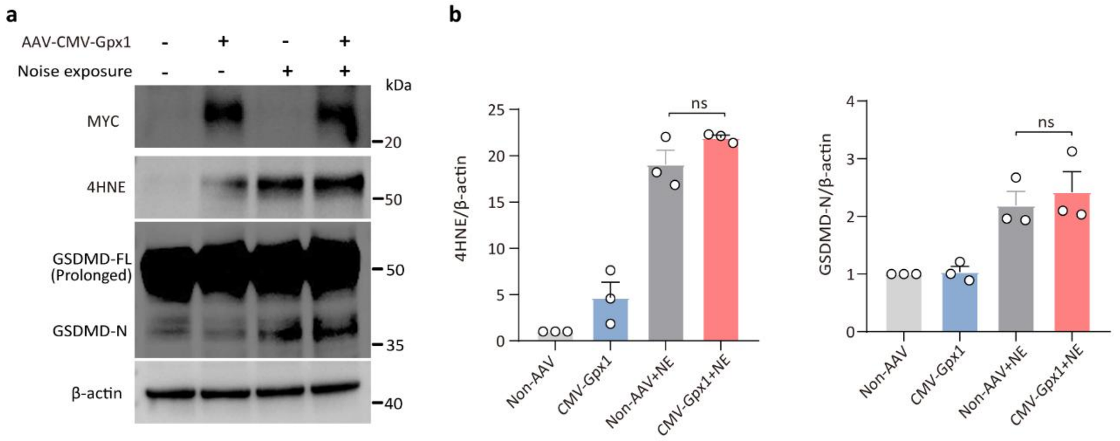

Supplementary Fig. 8: GPX1 overexpression fails to attenuates noise-induced oxidative stress and GSDMD cleavage

a WB analysis of the indicated proteins in cochlear tissues from non-AAV- and AAV-CMV-*Gpx1*-injected mice, with/without noise exposure. Cochleae were harvested at 8 h-PNE. The MYC band confirms AAV-mediated overexpression of GPX1 in the cochlea. Detection parameters included short exposure (5 s) for GSDMD-FL and prolonged exposure (30 s) for GSDMD-N to ensure optimal band visualization. **b** Quantitative analysis of target bands intensities across groups. All data are presented as mean ± SEM (n = 3 replicates, with each replicate representing a pooled sample of four cochleae). Statistical analysis was performed using one-way ANOVA with Bonferroni post hoc test. ns, not significant.

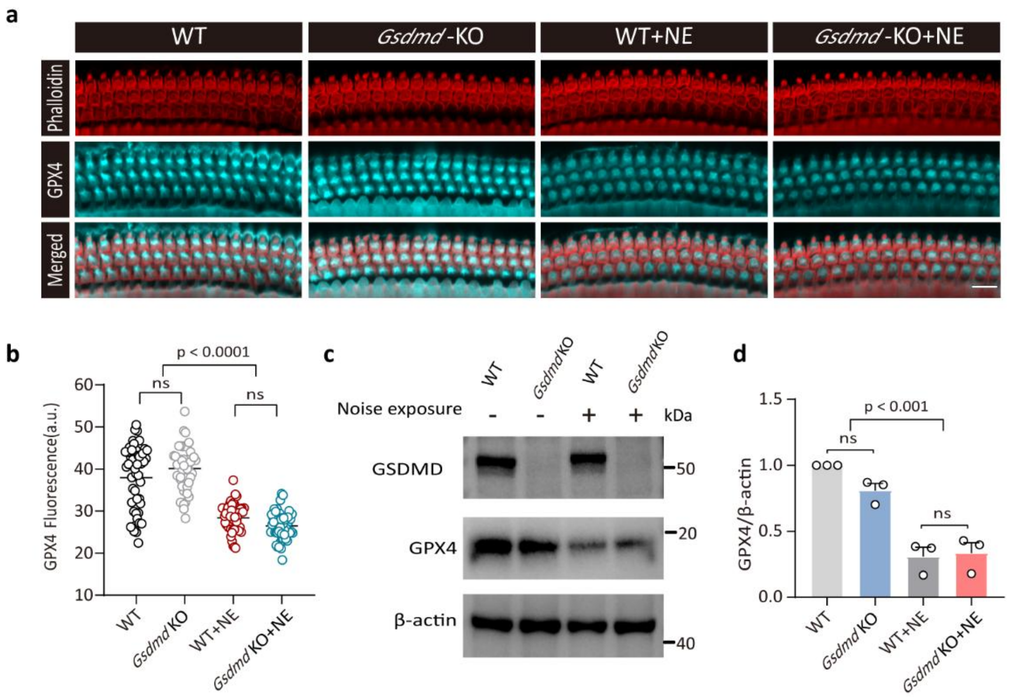

Supplementary Fig. 9: Knocking out *Gsdmd* fails to prevent noise-induced GPX4 downregulation.

a, b Immunolabeling analysis of GPX4 (cyan) in outer hair cells (OHCs) from WT and *Gsdmd* KO mice with/without noise exposure (scale bar = 10 μm). Cochleae were collected at 2 h post-noise exposure. The analysis focused on the 22.6-32 kHz frequency region. Circles in panel **b** represents individual OHCs. The confocal plane showing cytoplasm near the cuticular plate of OHCs was confirmed by phalloidin (red) staining. **c d** WB analysis of cochlear GPX4 level in WT and *Gsdmd* KO mice(6-week-old) exposed to noise or not. Data are presented as mean ± SEM (n = 3 biological replicates). Statistical analyses were conducted one-way ANOVA with Bonferroni's post hoc test. ns, not significant. NE: noise exposure.

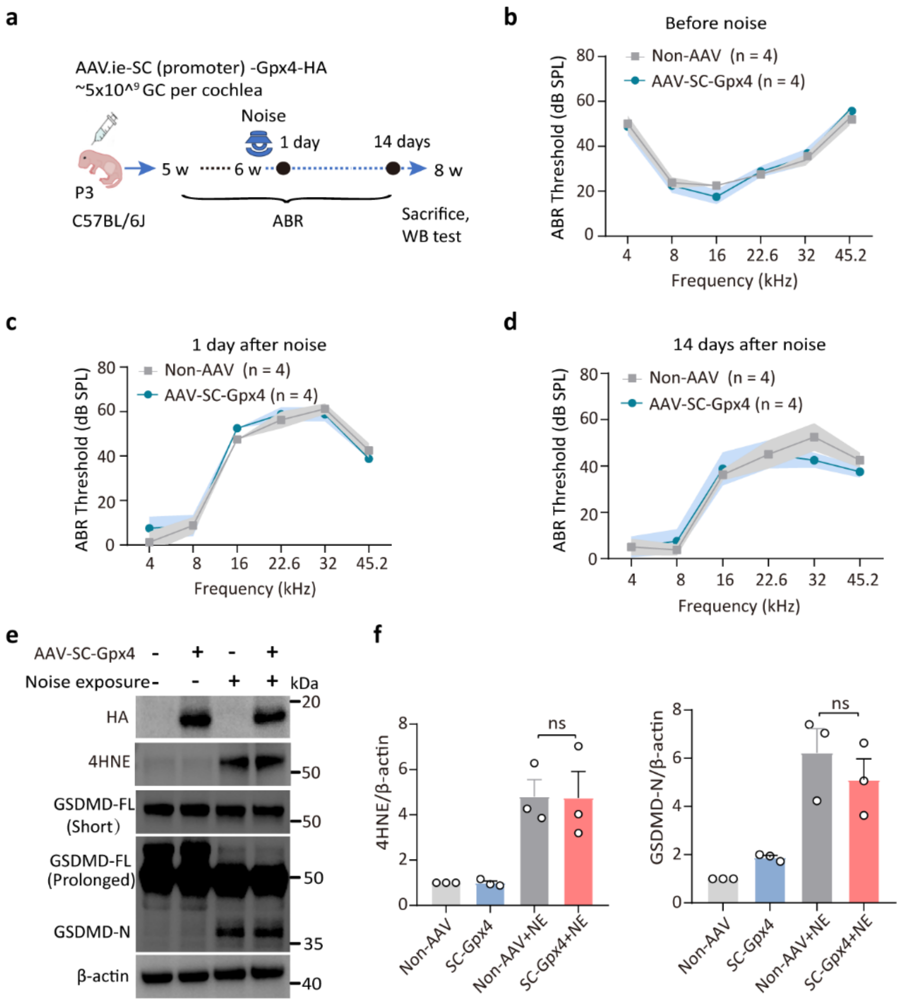

Supplementary Fig. 10: GPX4 overexpression in SCs fails to prevent noise-induced hearing dysfunction and GSDMD cleavage.

a Schematic of the experimental design. AAV.ie-SC-*Gpx4* (5 x 10^9 GCs) was injected into P3 C57BL/6J mice, followed by auditory electrophysiology and cochlear morphologic analysis. **b** Baseline ABR thresholds of non-AAV (gray) and AAV-SC-*Gpx4* (cyan) groups before noise exposure (n = 4)**. c, d** ABR threshold shifts non-AAV (gray) and AAV-SC-*Gpx4* (cyan) mice at 1 day and 14 days post-noise exposure. **e, f** Immunoblot analysis of 4-HNE and GSDMD-N levels in cochleae from non-AAV- and AAV-SC-*Gpx4*-injected mice with/without noise exposure. Cochleae were collected at 8 h-PNE. Detection parameters with short exposure (5 s) and prolonged exposure (30

s) to visualize GSDMD-FL and GSDMD-N bands, respectively. Data are presented as mean ± SEM (n = 3 biological replicates). Statistical analyses were conducted using two-way (**b-d**) and one-way (**f**) ANOVA with Bonferroni's post hoc test. ns, not significant (p value > 0.05). SC: supporting cell. GC: genome copies. NE: noise exposure. PNE: post-noise exposure.

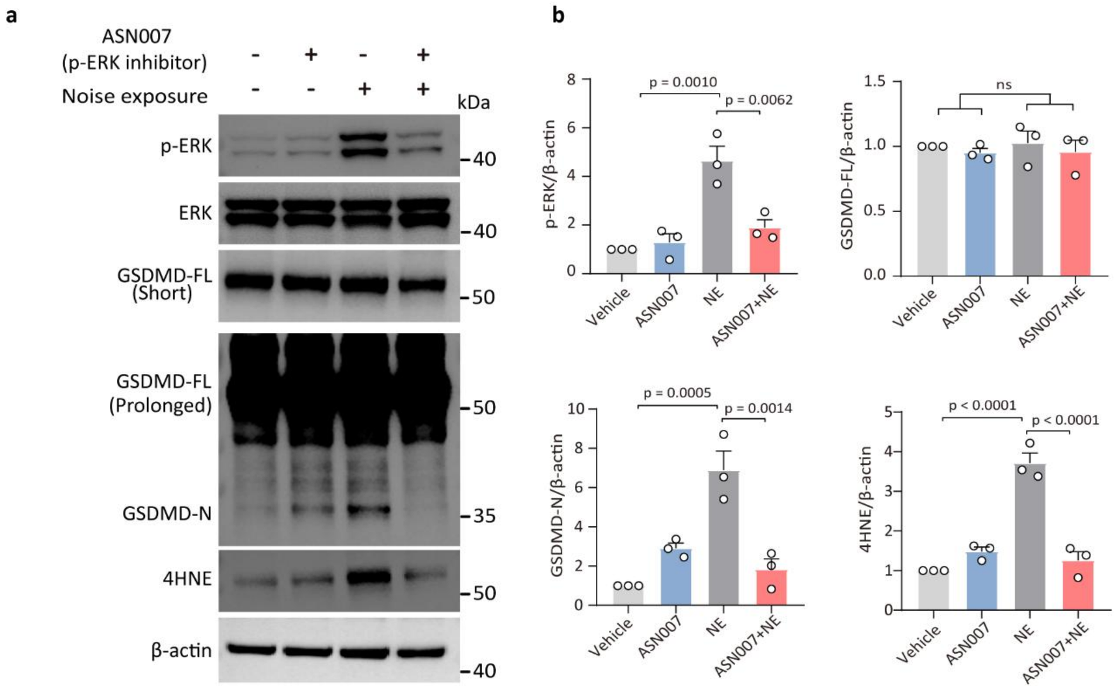

Supplementary Fig. 11: Pharmacological inhibition of ERK phosphorylation attenuates noise-induced GSDMD activation

a, b Effect of ASN007 (an inhibitor to reduce p-ERK levels) on GSDMD, 4HNE, p-ERK, and t-ERK levels in WT mice after NE, evaluated at 8 h-PNE. Detection parameters included short exposure (5 s) and prolonged exposure (30 s) to visualize GSDMD-FL and GSDMD-N bands, respectively. Data are presented as mean ± SEM (n = 3 biological replicates). Statistical analyses were conducted using one-way ANOVA with Bonferroni's post hoc test. ns, not significant (p value > 0.05). NE: noise

exposure.

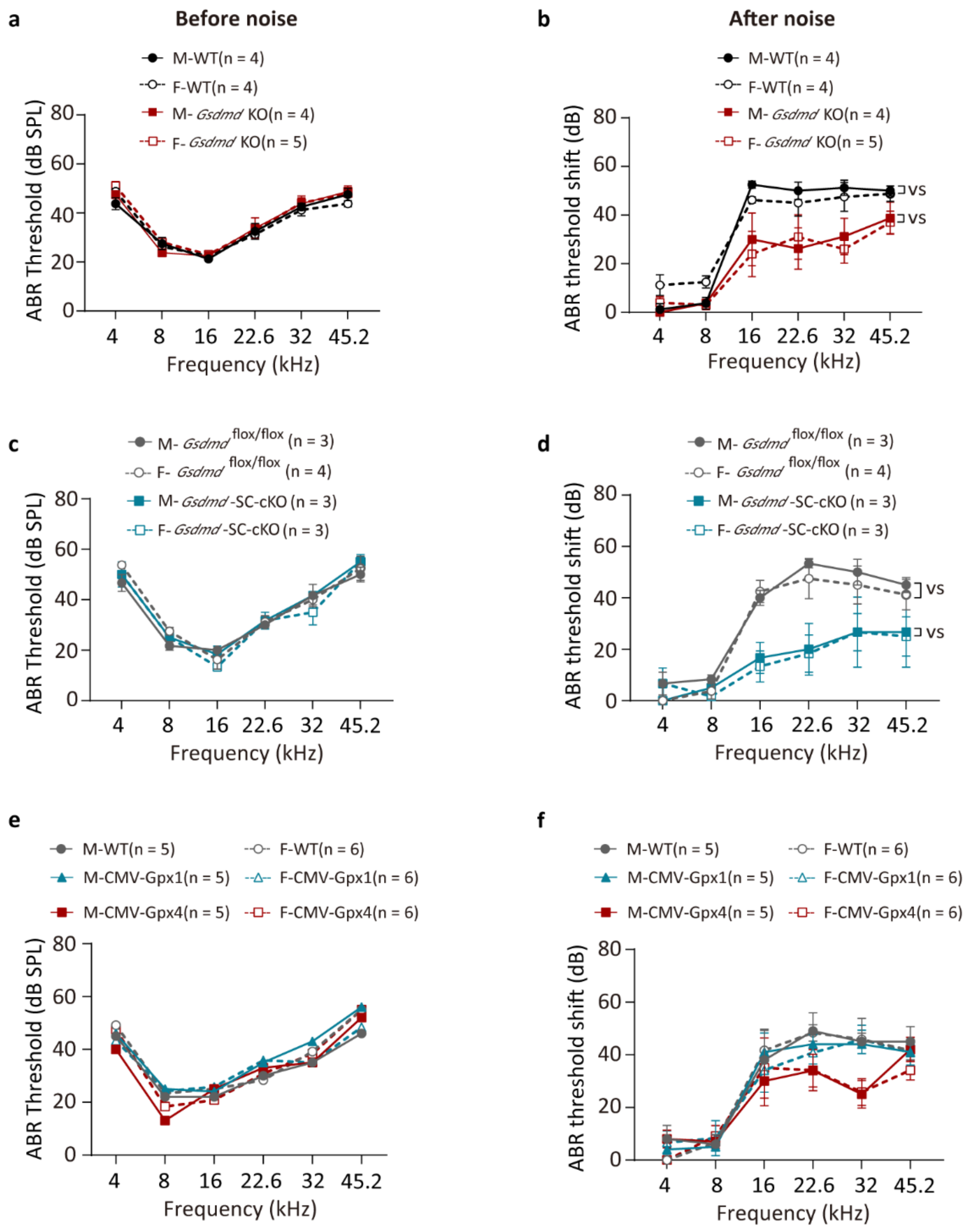

Supplementary Fig. 12: Sex-stratified analyses for auditory functional phenotypes in specific gene manipulated mice.

Baseline ABR thresholds showed no significant differences between male (solid lines) and female (dashed lines) mice in *Gsdmd*-KO (**a**), *Gsdmd*-SC-cKO (**c**), or AAV-CMV-*Gpx1*/*Gpx4* (**e**) groups, respectively. Similarly, ABR threshold shifts at 14 days post-noise exposure (14 d-PNE) revealed no sex differences in susceptibility to noise-

induced hearing loss across these cohorts (**b**,**d** and **f**). Data are presented as mean ± SEM. Statistical analyses were conducted using two-way ANOVA with Bonferroni's post hoc test.

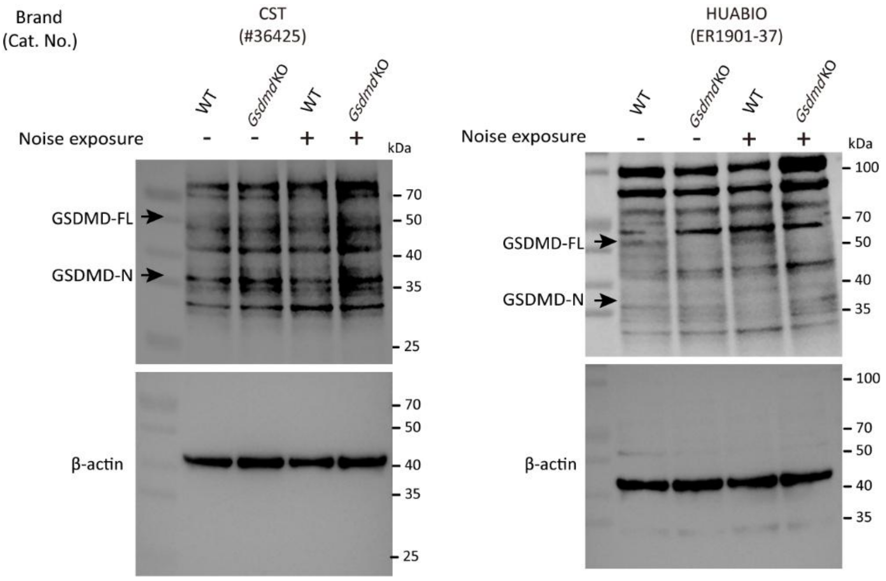

Supplementary Fig. 13: Validation of GSDMD-N fragment antibodies.

Commercially available GSDMD-N antibodies showed non-specific binding in cochlear tissue and failed validation in *Gsdmd*-KO mice.

Table 1: Primer information used for mouse genotyping

Gene	Primer sequence 5'-3'
Gsdmd KO (T010437)	F1: AAGCCCAGCCTGCCATCTAAC<br>R1: TGTTCTCAGGCTGCTGAAGGTG
	F2: TCTCTGATGTCGTCGATGGGAA<br>R2: GCTGTTGTTTCTACTACTCCACTCCT
Gsdmd $^{flox/flox}$ (T059954)	F1: GCAGTATCTCTCAGCACTGAATGAGG<br>R1: AAGGCTAGGGAATCCAGGAAGTCT
	F2: AATGCACTGAGATCCAGAAGTTGG<br>R2: GTCATGGTGGCACACCATAACA
Casp 1 KO	P1: GAGACATATAAGGGAGAAGGG<br>P2: ATGGCACACCACAGATATCGG
	P3: TGCTAAAGCGCATGCTCCAGACTG<br>P4: ATGGCACACCACAGATATCGG
Casp1/11 DKO	Casp1-WT-F: GAAGAGATGTTACAGAAGCC<br>Casp1-WT-R: CATGCCTGAATAATGATCACC<br>Casp1-KO-R: GCGCCTCCCCTACCCGG
	Casp11F: GACCCTATGACTTATTCATCTTCTG<br>Casp11-R: CTGCAAAGCATTCACCTTCA
Tlr4 KO	P1: AGCAAAGACAAGGGAGTAAGAA<br>P2: GCCTGAAATACTGGCTAAAAG
	P3: GTCCCTGATGACATTCCTTCT<br>P4: CTGTTTCTTGCCCATAGTTGA

Table 2: List of primary antibodies

Primary antibody	Antibody isotype	Source	IF or 1:100 (WB) Dilution
GSDMD	Rabbit	Abcam (ab219800)	1:200(1:1000)
GSDMD	Rabbit	Abcam (ab209845)	1:200 or 1:100 (1:1000)
GSDMDC1	Rabbit	Novus Bio (NBP2-33422)	1:200(1:1000)
GSDMD	Mouse IgG1	Abnova (H00079792-M01)	1:200(1:1000)
GSDMDC1	Mouse IgG1	Santa (sc-393656)	1:200(1:1000)
αII-Spectrin	Mouse lgG2b	BioLegend (SIG-39702)	1:200
MyosinVIIa	Rabbit	Proteus Biosciences (25-6790)	1:200
MYO7A 138-1	Mouse IgG1	DSHB	1:20
Cre Recombinase	Rabbit	CST (D7L7L, 15036S)	1:200 (1:1000)
Ctbp2	Mouse lgG1	BD Biosciences (612044)	1:200
IL-1β	Rabbit	CST (D6D6T, #31202)	(1:1000)
CASP11	Rat	Novus Bio (NB120-10454)	(1:1000)
CASP1	Mouse lgG1	Santa Cruz P20 (sc-398715)	(1:100)
4HNE	Mouse IgG2b	R&D Systems (MAB3249)	1:100(1:1000)
GPX1	Rabbit	Abcam (ab22604)	(1:1000)
GPX4	Rabbit	Abcam (ab125066)	1:200 (1:1000)
MYC	Mouse	CST (9B11, #2276)	(1:1000)
HA	Rabbit	CST (C29F4)	1:200 (1:1000)
p-ERK1/2	Rabbit	CST (D13.14.4E, XP, #4370)	1:200 (1:1000)
ERK1/2	Rabbit	CST (137F5, #4695)	(1:1000)
β-actin	Rabbit	ABclonal (AC038)	1:50000
GSDMD-N	Rabbit	CST (E7H9G, #36425)	(1:1000)
GSDMD-N	Rabbit	HUABIO (ER1901-37)	(1:1000)
