# Supplementary material for: Supporting cells orchestrate noise-induced hearing loss via a Gasdermin D-dependent signaling loop with hair cells
Source: Nat Commun. 2025 Dec 17;16:11181. doi: 10.1038/s41467-025-66152-6 (PMC12712061; doi:10.1038/s41467-025-66152-6)
Supplement: Supplementary file 2 — Reporting summary [file 41467_2025_66152_MOESM2_ESM.pdf]

Corresponding author(s): Zhen Zhang
Last updated by author(s): Jul 28, 2025

# Reporting Summary

Nature Portfolio wishes to improve the reproducibility of the work that we publish. This form provides structure for consistency and transparency in reporting. For further information on Nature Portfolio policies, see our Editorial Policies and the Editorial Policy Checklist.

## Statistics

For all statistical analyses, confirm that the following items are present in the figure legend, table legend, main text, or Methods section.

| n/a | Confirmed | |
|---|---|---|
| ☐ | ☒ | The exact sample size (*n*) for each experimental group/condition, given as a discrete number and unit of measurement |
| ☐ | ☒ | A statement on whether measurements were taken from distinct samples or whether the same sample was measured repeatedly |
| ☐ | ☒ | The statistical test(s) used AND whether they are one- or two-sided<br>*Only common tests should be described solely by name; describe more complex techniques in the Methods section.* |
| ☒ | ☐ | A description of all covariates tested |
| ☐ | ☒ | A description of any assumptions or corrections, such as tests of normality and adjustment for multiple comparisons |
| ☐ | ☒ | A full description of the statistical parameters including central tendency (e.g. means) or other basic estimates (e.g. regression coefficient) AND variation (e.g. standard deviation) or associated estimates of uncertainty (e.g. confidence intervals) |
| ☐ | ☒ | For null hypothesis testing, the test statistic (e.g. *F*, *t*, *r*) with confidence intervals, effect sizes, degrees of freedom and *P* value noted<br>*Give P values as exact values whenever suitable.* |
| ☒ | ☐ | For Bayesian analysis, information on the choice of priors and Markov chain Monte Carlo settings |
| ☒ | ☐ | For hierarchical and complex designs, identification of the appropriate level for tests and full reporting of outcomes |
| ☐ | ☒ | Estimates of effect sizes (e.g. Cohen's *d*, Pearson's *r*), indicating how they were calculated |

*Our web collection on statistics for biologists contains articles on many of the points above.*

## Software and code

Policy information about availability of computer code

| | |
|---|---|
| Data collection | The RZ6 BioAMP Processor (TDT, USA); Confocal laser scanning microscopy (LSM 710 META; Zeiss, Shanghai, China); Leica Stellaris 5 confocal microscope; ImageJ software (National Institutes of Health, Bethesda, MD, USA); Panoramic scanner(3DHISTECH); Tanon 5200 Imaging System (Tanon, Shanghai, China). |
| Data analysis | The RZ6 system (TDT) software; GraphPad Prism 8 (GraphPad Software, San Diego, 705 CA, USA); Adobe Illustrator; ImageJ software ; CaseViewer. |

For manuscripts utilizing custom algorithms or software that are central to the research but not yet described in published literature, software must be made available to editors and reviewers. We strongly encourage code deposition in a community repository (e.g. GitHub). See the Nature Portfolio guidelines for submitting code & software for further information.

## Data

Policy information about availability of data

All manuscripts must include a data availability statement. This statement should provide the following information, where applicable:

- Accession codes, unique identifiers, or web links for publicly available datasets
- A description of any restrictions on data availability
- For clinical datasets or third party data, please ensure that the statement adheres to our policy

Datasets generated during and analyzed in this study are available from the corresponding author upon reasonable request. Source data are provided with this article.

## Research involving human participants, their data, or biological material

Policy information about studies with human participants or human data. See also policy information about sex, gender (identity/presentation), and sexual orientation and race, ethnicity and racism.

| | |
|---|---|
| Reporting on sex and gender | No Reporting on sex and gender was referenced in this manuscript |
| Reporting on race, ethnicity, or other socially relevant groupings | No Reporting on race, ethnicity,or other socially relevant groupings |
| Population characteristics | N/A |
| Recruitment | N/A |
| Ethics oversight | N/A |

Note that full information on the approval of the study protocol must also be provided in the manuscript.

# Field-specific reporting

Please select the one below that is the best fit for your research. If you are not sure, read the appropriate sections before making your selection.

☒ Life sciences ☐ Behavioural & social sciences ☐ Ecological, evolutionary & environmental sciences

For a reference copy of the document with all sections, see nature.com/documents/nr-reporting-summary-flat.pdf

# Life sciences study design

All studies must disclose on these points even when the disclosure is negative.

| | |
|---|---|
| Sample size | For in vivo experiments, cohort size was determined by the types of experiment and the availability of animals, since littermates were used in each experiment. Our sample size, ranging from 4 to 11 mice per group, are standard for cohort size in this field. |
| Data exclusions | No data were excluded. |
| Replication | All experiments were performed with at least three independent replicates , as indicated in the figure legends. |
| Randomization | All animals were randomized into groups at the start of the study. |
| Blinding | No blinding was used as it was not applicable for the nature of the experimental setup. |

# Reporting for specific materials, systems and methods

We require information from authors about some types of materials, experimental systems and methods used in many studies. Here, indicate whether each material, system or method listed is relevant to your study. If you are not sure if a list item applies to your research, read the appropriate section before selecting a response.

## Materials & experimental systems

| n/a | Involved in the study | |
|---|---|---|
| ☐ | ☒ | Antibodies |
| ☒ | ☐ | Eukaryotic cell lines |
| ☒ | ☐ | Palaeontology and archaeology |
| ☐ | ☒ | Animals and other organisms |
| ☒ | ☐ | Clinical data |
| ☒ | ☐ | Dual use research of concern |
| ☒ | ☐ | Plants |

## Methods

| n/a | Involved in the study | |
|---|---|---|
| ☒ | ☐ | ChIP-seq |
| ☒ | ☐ | Flow cytometry |
| ☒ | ☐ | MRI-based neuroimaging |

# Antibodies

**Antibodies used**

All antibody details including clone and manufacturer information are included in Supplementary Materials Table S1 and also listed below:
1. Rabbit GSDMD antibody,Abcam (ab219800, IF 1:200, WB 1:1000)
2. Rabbit GSDMD antibody,Abcam (ab209845, IF 1:200, IHC 1:100, WB 1:1000)
3. Rabbit GSDMDC1 antibody, Novus Bio (NBP2-33422, IF 1:200, WB 1:1000)
4. Mouse GSDMD antibody, Abnova (H00079792-M01, IF 1:200, WB 1:1000)
5. Mouse GSDMDC1 antibody, Santa (sc-393656, IF 1:200, WB 1:1000)
6. Mouse α□-Spectrin antibody, BioLegend (SIG-39702, IF 1:200)
7. Rabbit Myosin?a antibody, Proteus Biosciences (25-6790, IF 1:200)
8. Mouse MYO7A 138-1 antibody, DSHB (IF 1:20)
9. Rabbit Cre Recombinase antibody, CST (D7L7L, 15036S, IF 1:200, WB 1:1000)
10. Mouse Ctbp2 antibody, BD Biosciences (612044, IF 1:200)
11. Rabbit IL-1β antibody, CST (D6D6T, #31202, WB 1:1000)
12. Rat CASP11 antibody, Novus Bio (NB120-10454, WB 1:1000)
13. Mouse CASP1 antibody, Santa Cruz P20 (sc-398715, WB 1:1000)
14. Mouse 4HNE antibody, R&D Systems (MAB3249, IF 1:200, WB 1:1000)
15. Rabbit GPX1 antibody, Abcam (ab22604, WB 1:1000)
16. Rabbit GPX4 antibody, Abcam (ab125066, IF 1:200, WB 1:1000)
17. Mouse MYC antibody, CST (9B11, #2276, WB 1:1000)
18. Rabbit HA antibody, CST (C29F4, IF 1:200, WB 1:1000)
19. Rabbit p-ERK1/2 antibody, CST (D13.14.4E, XP, #4370, IF 1:200, WB 1:1000)
20. Rabbit ERK1/2 antibody, CST (137F5, #4695, WB 1:1000)
21. Rabbit β-actin antibody, ABclonal (AC038, WB 1:1000)
22. Rabbit GSDMD-N antibody, CST (E7H9G, #36425, WB 1:1000)
23. Rabbit GSDMD-N antibody, HUABIO (ER1901-37, WB 1:1000 )

**Validation**

All antibodies used in this study have been commercially available . The complete information and validation information for each antibody as well as previous publications that have used each antibody can be found on the manufacturer's website.

# Animals and other research organisms

Policy information about studies involving animals; ARRIVE guidelines recommended for reporting animal research, and Sex and Gender in Research

**Laboratory animals**

All animals were performed using adult male and female mice (5-6 w) as indicated for each experiments. All mouse strains were backcrossed to the C57BL/6 for at least 10 generations.
C57BL/6J mice (SBF Biotechnology Laboratory Animal Field); Gsdmd KO mice (GemPharmatech,T010437); Gsdmd flox/flox (GemPharmatech,T059954); B6/JGpt-H11 em1Cin (CAG-LoxP-ZsGreen-Stop-LoxP-tdTomato)/Gpt reporter mice (GemPharmatech, T006163); Tlr4 KO mice (Shanghai Model Organisms Center, NM-KO-18052) ; Casp1 KO mice (Cyagen Biosciences, 12362) ; Casp1/11 DKO mice were kindly provided by Prof. Haibing Zhang (Shanghai Institute of Nutrition and Health, Chinese Academy of Sciences, Shanghai, China); Casp11 KO mice were generously provided by Prof. Feng Shao (National Institute of Biological Sciences, Beijing).
Mice were housed in a temperature-controlled facility (24 °C) under a 12-hour light/dark cycle with free access to standard laboratory chow and sterilized water.

**Wild animals**

No wild animals were used in this study

**Reporting on sex**

Male and female mice were analyzed together in the main study. As for evaluation of auditory function, sex-based hearing differences were assessed separately, as detailed in Supplementary Materials Extended Data Fig. S10.

**Field-collected samples**

No field collected samples were used in this study

**Ethics oversight**

All animal procedures were approved by the Ethics Committee of Shanghai Sixth People's Hospital affiliated to Shanghai Jiao Tong University School of Medicine (DWLL2023-0261).

Note that full information on the approval of the study protocol must also be provided in the manuscript.

## Plants

| | |
|---|---|
| Seed stocks | N/A |
| Novel plant genotypes | N/A |
| Authentication | N/A |
